# Tuberculoid leprosy: An *in vivo* microvascular evaluation of cutaneous lesions

**DOI:** 10.1371/journal.pone.0227654

**Published:** 2020-01-13

**Authors:** Livia Pino, Maria das Graças Coelho de Souza, Omar Lupi, Eliete Bouskela

**Affiliations:** 1 Laboratório de Pesquisas Clínicas e Experimentais em Biologia Vascular, Centro Biomédico, Universidade do Estado do Rio de Janeiro (UERJ), Rio de Janeiro, Rio de Janeiro, Brazil; 2 Departamento de Dermatologia, Universidade Federal do Estado do Rio de Janeiro (UniRio), Rio de Janeiro, Rio de Janeiro, Brazil; National Taiwan University Hospital, TAIWAN

## Abstract

Tuberculoid leprosy (TT) is characterized by cutaneous lesions called plaques. Although microvascular ultrastructure of TT patients’ skin is well-documented, little is known about functional aspects of their microcirculation. We aimed, for the first time, to evaluate, *in vivo*, the microcirculation of TT cutaneous lesions. Seven TT patients, males, under treatment were included in the study. The spectral analysis of frequency components of flowmotion (endothelial, sympathetic, myogenic, cardiac and respiratory) was performed using laser Doppler flowmetry (LDF). Endothelial dependent and independent vasodilatations were assessed by LDF associated to acetylcholine (ACh) and sodium nitroprusside (SNP) iontophoresis, respectively. Vessel density (VD), perfused vessel density (PVD), proportion of perfused vessels (PPV%), microvascular flow index (MFI) and flow heterogeneity index (FHI), reflecting tissue perfusion and oxygenation, were evaluated through sidestream dark field (SDF) imaging. All microvascular analysis were performed in TT lesions and in healthy skin in the contralateral limb of the same patient, used as control skin. VD, PVD and PPV% and MFI were significantly lower in the cutaneous lesion compared to contralateral healthy skin. The contribution of different frequency components of flowmotion, endothelial dependent and independent vasodilatations and FHI were not statistically different between control skin and cutaneous lesion. Our results suggest that TT cutaneous lesions have a significant impairment of tissue perfusion, which may aggravate peripheral nerve degeneration caused by *Mycobacterium leprae* infection.

## Introduction

Leprosy is a chronic granulomatous infection of skin and peripheral nerves caused by *Mycobacterium leprae* (*M*. *leprae*) [1]. This obligatory intracellular pathogen causes nerve damage, affecting sensory, motor and autonomic fibers which results in disabilities and deformities [2,3]. Leprosy is characterized by a clinical pathological spectrum, based on host immune response [4]. The immunopathologic spectrum of leprosy encompasses five forms: tuberculoid-tuberculoid (TT), borderline tuberculoid, borderline borderline, borderline lepromatous and lepromatous leprosy (LL) [5].

The hallmark of TT is the presence of one to three cutaneous lesions in a patient, called plaques. These lesions are uniformly circular or oval, erythematous/hypopigmented, hairless, scaly, dry and anesthetic [3].

Ultrastructural observations of skin biopsies from TT patients have demonstrated that cutaneous and endoneural vessels present hypertrophied endothelial cells [6,7]. Endothelial cell hypertrophy was of such intensity that it blocked the capillary lumen [7] and it is due to an extensive rough endoplasmic reticulum [6], which is characteristic of increased protein synthesis and metabolic activity typical of chronic inflammatory states and immune reactions [8]. Additionally, the basement membrane of endothelial cells presented lamellary thickening associated with increased number of leukocytes around blood vessels [6]. To our knowledge, the functional aspects of TT cutaneous lesion microcirculation have not been investigated yet.

The microcirculation encompasses vessels with diameters up to 100 μm (arterioles, capillaries, venules and microlymphatics) and is responsible for oxygen and nutrients delivery to tissues, cell waste withdrawal and peripheral vascular resistance regulation [9,10].

Arterioles display spontaneous rhythmic variations of diameter, called vasomotion that elicits blood flow oscillations, termed flowmotion, and ensures an intermittent, but adequate, blood flow distribution to tissues [11–13].

Assessment of human vasomotion and its consequent flowmotion is possible by means of spectral analysis of laser Doppler flowmetry (LDF) signal [12]. LDF can also be used to investigate endothelial microvascular function when associated to iontophoresis of endothelial -dependent and -independent vasodilators: acetylcholine (ACh), and sodium nitroprusside (SNP), respectively. Iontophoresis allows transdermal delivery of these vasodilators using a weak current and the subsequent elevation of blood flow is, then, recorded by the LDF apparatus [14,15].

Sidestream dark field (SDF) consists in a simple and non-invasive imaging device that provides well-defined images of the microcirculation [16]. With this system it is possible to observe the number of capillaries with flowing red blood cells and therefore estimate tissue blood perfusion as well as the heterogeneity of blood flow.

Thus, we have aimed, for the first time, to evaluate, *in vivo*, microvascular alterations of skin lesions from TT patients using laser Doppler flowmetry (associated or not to iontophoresis) and sidestream dark field (SDF) imaging.

## Materials and methods

This is a cross-sectional study approved by the local Ethics Committee (Ethics Committee of Universidade do Estado do Rio de Janeiro, COEP 0077/2011 registration no. 060.3.2011, performed according to principles outlined in the Declaration of Helsinki. This study was registered at Brazilian Register of Clinical Trials (Registro Brasileiro de Ensaios Clínicos, ReBEC) number RBR-2vz89f, Trial URL: http://www.ensaiosclinicos.gov.br/rg/RBR-2vz89f/.

### Subjects

Seven TT patients at the beginning of treatment (just after the diagnostic of the disease) were included in the study. Microvascular variables of skin lesions of these patients were compared to symmetrical healthy skin in the contralateral limb, used as control. All participants of the study have met the following inclusion criteria and have signed the written informed consent.

#### Inclusion criteria

Men with recent diagnostic of TT, at the beginning of treatment, with ages between 20 and 60 years old, body mass index (BMI) between 18 and 29.9 kg/m^2^, Fitzpatrick’s phototypes I to IV. The Fitzpatrick skin phototype is a classification commonly used to describe the individual’s skin type in terms of response to ultraviolet radiation exposure [17]. Fitzpatrick’s phototype I refers to white unexposed skin that sunburns, but never tans), II refers to white unexposed skin that sunburns and tans minimally, III refers to white unexposed skin that sunburns and tans and IV refers to white unexposed skin that rarely sunburns and tans with ease) [17], able to follow given directions and to attend microvascular assessments were included in the study.

#### Exclusion criteria

Women or men with previously confirmed diagnostic of hypertension, diabetes mellitus, BMI ≥30 kg/m^2^. Individuals with darker phototypes (since skin pigmentation makes the evaluation of cutaneous microcirculation very difficult or even impossible): Fitzpatrick’s phototypes V (which refers to brown unexposed skin that rarely sunburns and always tans) and VI (which refers to black unexposed skin that never sunburns and always tans, totally pigmented) [17]. Past or present history of smoking and ages under 20 and over 60 years old were excluded.

#### Study recruitment

Initially 22 patients, who met the inclusion criteria, were recruited for the study in the ambulatory Souza Araújo from Fundação Oswaldo Cruz (Fiocruz). From these total, 13 patients did not attend the examinations and two were excluded because of their cutaneous phototype (Fitzpatrick’s phototype V). Thus, only seven patients underwent the examinations proposed in the study protocol.

### Microvascular assessment

All participants were asked to arrive in the laboratory after 12 h overnight fast and to abstain from caffeine and alcohol during the last 24 hours. They were accommodated in an acclimatized room (23±1 °C) during 20 minutes before microvascular evaluations. All subjects had their anthropometric variables assessed and blood pressure evaluated before the examination to ensure that they met the inclusion criteria.

### Spectral analysis of flowmotion

Skin blood perfusion and flowmotion were evaluated by LDF apparatus (PeriFlux System PF5000, Perimed AB, Stockholm, Sweden) consisting of a transmission of low-power laser light (780 nm) to the tissue though a fiber optic probe that penetrates 0.4–1.0 nm. The light penetration allows the assessment of net red blood cell flow in arbitrary perfusion units (PU) that corresponds to the concentration of moving blood cells and their velocity, in arterioles, capillaries and venules and in anastomosis of deeper blood vessels in dermal layers, within an area of 1mm^2^ [18]. The LDF signal was recorded continuously during 20 min by an interfaced computer equipped with Perisoft software (PSW 2.50, Perimed AB, Stockholm, Sweden) in order to assess skin blood flow and vasomotion. For these measurements, the LDF probe was placed in the central area of the cutaneous lesion as well as the central of the skin area symmetrical to the lesion in the contralateral limb (healthy control area) of TT patients.

For analysis of LDF signal we used fast Fourier transform (Perisoft software, PSW version 2.50, Perimed AB, Stockholm, Sweden). This analysis determines the contribution of different frequency components of flowmotion through the variability of LDF signal. The frequency spectrum between 0.01 and 1.6 Hz was divided into five frequency intervals: endothelial (0.01–0.02 Hz), sympathetic (0.02–0.06 Hz), myogenic, related to vascular smooth muscle cell (VSMC) activity (0.06–0.15 Hz), respiratory (0.15–0.4 Hz) and cardiac, associated to heart beat frequency (0.4–1.6 Hz) [19,20]. Mean total amplitude value of the total spectrum as well as the mean amplitude values of each frequency interval were recorded and normalized (absolute amplitude at a particular frequency interval divided by the mean amplitude of the entire spectrum) [21]. The normalized results of skin lesions were then compared to those observed in the control skin of TT patients.

### Iontophoresis of acetylcholine and sodium nitroprusside

Endothelium-dependent and -independent vasodilatations were evaluated by LDF combined to iontophoresis of ACh and SNP, respectively. This protocol was adapted from [18,22], since we needed to perform iontophoresis of ACh and SNP exactly at the same sites (surface of the skin lesion or the surface of control skin in the contralateral limb). In general, different sites were used for ACh and SNP iontophoresis. As ACh has a short half-life, it was used first. The SNP iontophoresis was started 15 min after the end of ACh iontophoresis in order to avoid any possible interference of ACh on SNP iontophoresis.

ACh (Acetylcholine, Sigma-Aldrich, Saint Louis, MO, USA) solution at 1% was delivered by nine iontophoretic pulses of 0.1 mA during 20 s with a 60 s interval between two successive pulses on cutaneous lesion and on symmetrical healthy skin in the contralateral limb, using an anodal current. Similarly, SNP (sodium nitroprusside, Niprid^®^ 10mg/ml–Biolab, São Paulo, Brazil) was delivered by seven iontophoretic pulses of 0.2 mA during 20 s with a 180 s interval between two successive pulses on cutaneous lesion and on symmetrical healthy skin in the contralateral limb, using a cathodal current.

During ACh and SNP iontophoresis, it was possible to evaluate cutaneous blood perfusion (in perfusion units–PUs) at baseline and plateau and vasodilatation expressed in absolute values (difference between plateau and baseline in PUs) and in percentage (% of increase from baseline to plateau). These values were compared between lesions and control skin of TT patients.

### Sidestream dark field (SDF) imaging assessment

After acclimatization, the microcirculation of skin lesion and contralateral healthy skin of each TT patient were assessed by SDF imaging (Microvision Medical B.V., Amsterdam, the Netherlands) at five different points, according to criteria recommended by De Backer [16].

Images were recorded for 10 s at each point and evaluated thereafter using the Automated Vascular Analysis (AVA 3.0; Microvision Medical B.V., Amsterdam, the Netherlands).

The score proposed by De Backer and coworkers [16] takes into account the principle that density of vessels is proportional to the number of vessels crossing arbitrary lines. In this score, three equidistant horizontal and three vertical lines are drawn in the screen monitor. The vessel density (VD), also known as De Backer score [23], was calculated as the number of vessels crossing the lines divided by total length of the lines. Additionally, the perfusion of vessels was classified into continuous (continuous flow for at least 20 s), absent (no flow for at least 20 s) and intermittent (at least 50% of the time with no flow) flow. The proportion of perfused vessels (PPV%) was calculated as 100 x (total number of vessels—vessels of intermittent or absence of flow) divided by total number of vessels. The perfused vessel density (PVD) is the product of VD and PPV and estimates the functional capillary density [16,23].

Due to its physiological relevance to oxygen exchange, small vessels (mostly capillaries) were separated from the large ones (predominantly venules) using a 20 μm cut-off [16].

For microvascular flow index (MFI) quantification, each image was divided into four quadrants and the circulation in each one was expressed in ordinal scale according to the predominant kind of flow: 0-no flow; 1 intermittent flow; 2-sluggish flow and 3-brisk flow of the skin lesions and control skin [24]. MFI represents the average score of all quadrants [23].

The flow heterogeneity index (FHI), which is the difference between the highest MFI and the lowest MFI divided by the mean MFI, was also calculated [24].

All these variables were evaluated in TT cutaneous lesion and in the skin area symmetrical to the lesion in the contralateral limb (healthy control skin) of TT patients.

### Statistical analysis

For all statistical analysis, GraphPad Prism^®^ 5 (GraphPad Software, Inc., San Diego, CA, USA) was used. Normal Gaussian distribution was checked using Shapiro-Wilk normality test. For parametric and non- parametric variables, data were expressed as mean±SD or mean±SEM and median [interquatile range], respectively. Inter group comparisons for parametric and non- parametric variables were performed by unpaired t-test and Mann Whitney U test, respectively. A *P* value of less than 0.05 was considered significant.

## Results

### Characteristics of TT patients

Table 1 presents anthropometric and clinical characteristics of TT patients. As expected, TT patients did not present obesity or hypertension.

**Table 1:** Clinical and anthropometrical characteristics of tuberculoid leprosy patients.

Variable	Mean ±SD
**Age (years)**	50.3±8.6
**Weight (kg)**	76.1±4.7
**Height (m)**	1.74±0.05
**BMI (kg/m**^**2**^**)**	25.2±1.8
**SBP (mmHg)**	123±10.55
**DBP (mmHg)**	77.6±7.58

BMI—body mass index, SBP—systolic blood pressure; DBP—diastolic blood pressure.

### Spectral analysis of flowmotion

**Fig 1:**
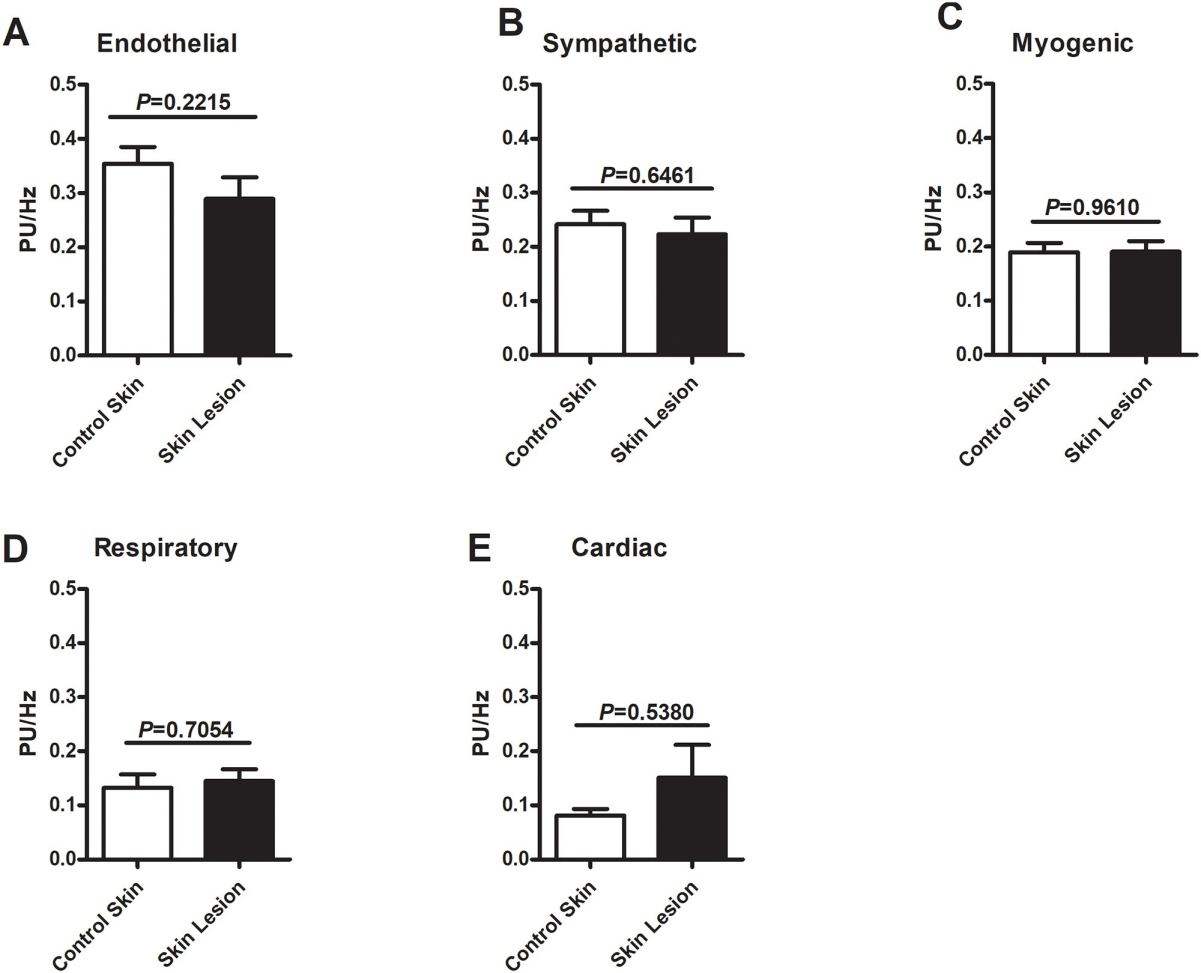
Spectral analysis of flowmotion. Comparison between different components of spectral analysis of flowmotion in Tuberculoid Leprosy (TT) cutaneous lesion and in the contralateral healthy limb (control) skin of the same patient: A) Endothelial; B) Sympathetic; C) Myogenic; D) Respiratory and; E) Cardiac components of flowmotion. Data were expressed as mean±SEM.

Spectral analysis of vasomotion frequency components (endothelial, sympathetic, myogenic, respiratory and cardiac) did not show any statistical difference between skin lesions and contralateral control skin. These results are shown on Fig 1.

### Iontophoresis of acetylcholine and sodium nitroprusside

During ACh and SNP iontophoresis, no statistical differences concerning blood flow at baseline and plateau were observed between skin lesions and healthy skin. The absolute and percentage values of blood flow, in these two skin areas, after ACh and SNP were not significantly different between cutaneous lesions and control skin. These results are depicted on Table 2.

**Table 2:** Microvascular measurements before and during iontophoresis using laser-Doppler flowmetry in healthy contralateral (control) skin and skin lesions of tuberculoid leprosy patients.

	Control Skin (n = 7)	Skin Lesions (n = 7)	*P* value
**ACh-mediated vasodilatation**			
**Baseline skin perfusion, PU**	14.38 [4.31–18.98]	8.42 [2.45–21.03]	0.8048
**Plateau, PU**	28.38 [19.86–53.2]	36.2 [11.17–62.48]	0.8048
**Number of doses to reach Plateau**	9.00[6.00–9.00]	8.00[5.00–9.00]	0.5921
**Absolute increase, PU**	16.20 [12.80–53.20]	27.80 [5.70–44.20]	0.9015
**Percentage increase, %**	190.60 [110.00–439.70]	242.00[125.40–441.90]	0.8048
**SNP-mediated vasodilatation**			
**Baseline skin perfusion, PU**	11.16 [7.99–13.11]	12.44[5.65–18.32]	0.9015
**Plateau, PU**	35.17 [27.57–41.38]	61.90 [27.11–80.62]	0.2593
**Number of doses to reach Plateau**	5.00[6.00–7.00]	7[7.00–7.00]	0.0699
**Absolute increase, PU**	26.20[17.50–29.30]	52.8 0[12.90–62.30]	0.6200
**Percentage increase, %**	258.50 [118.90–340.20]	340.10 [52.2–1042.00]	0.8048

Data are expressed as median [interquartile range]. ACh: acetylcholine. SNP: sodium nitroprusside. PU: perfusion units.

### Sidestream dark field (SDF) imaging assessment

**Fig 2:**
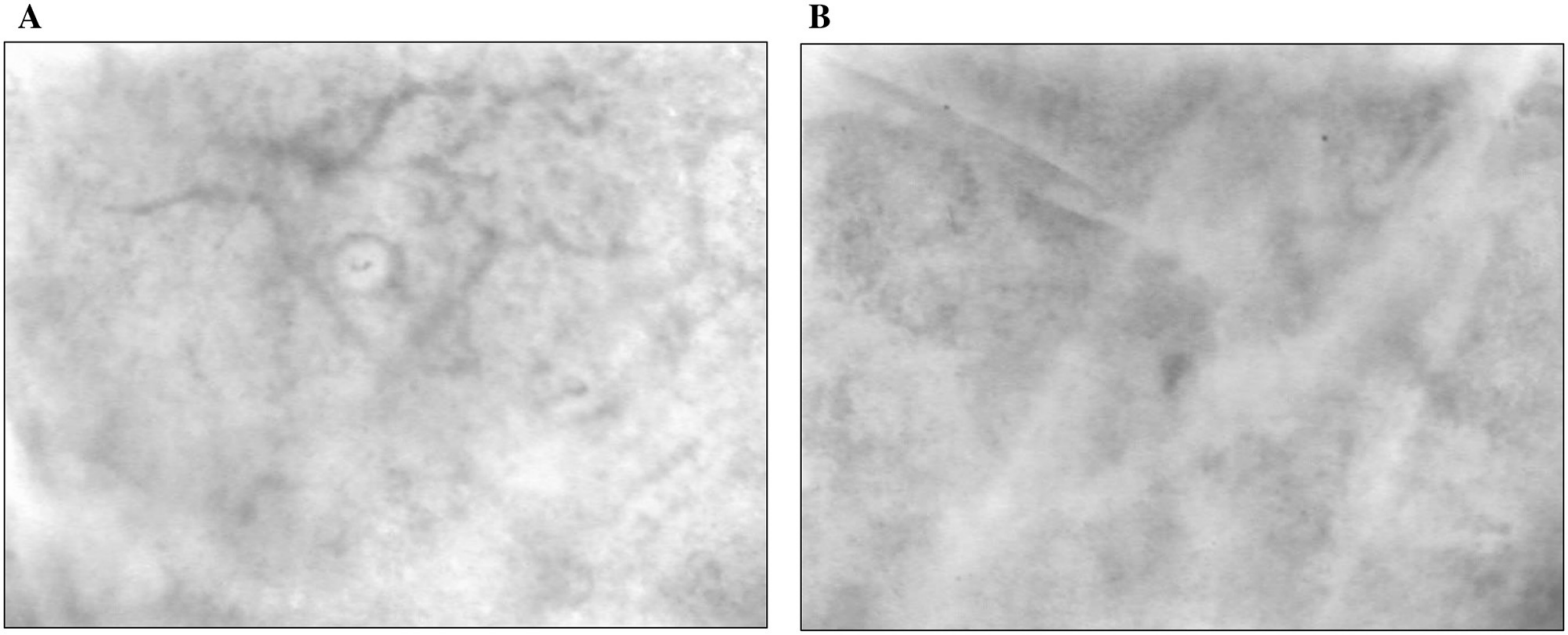
Sidestream dark field images of cutaneous microcirculation. Capillary blood perfusion in (A) normal skin (control) (B) Skin lesion (plaque) of the same tuberculoid leprosy patient.

Differences in capillary blood perfusion between normal and plaque skin are depicted on Fig 2.

VD, PVD and PPV% and MFI were significantly lower in the cutaneous lesion compared to healthy contralateral skin. On the other hand, FHI was not significantly different between skin lesion and control skin. These results are shown on Table 3.

**Table 3:** Comparison between cutaneous microcirculation of healthy contralateral (control) skin and skin lesions of tuberculoid leprosy patients using sidestream dark field imaging.

	Control Skin (n = 7)	Skin Lesion (n = 7)	*P* value
**VD (1/mm)**	17.49[17.08–18.42]	12.55[12.14–13.27]	0.0021
**PVD (1/mm)**	16.36[15.53–17.18]	10.29[10.19–11.11]	0.0006
**PPV (%)**	93.26[90.956–93.53]	83.33[82.86–86.40]	0.0006
**MFI (arbitrary units)**	2.500[2.250–2.500]	1.750[1.500–1.750]	0.0021
**FHI**	0.2069[0.00–0.2500]	0.1667[0.1579–0.3333]	0.3706

Data are expressed as median [interquartile range]. VD: vessel density. PVD: perfused vessel density. PPV: proportion of perfused vessels. MFI: microvascular flow index. FHI: flow heterogeneity index.

## Discussion

We have evaluated, for the first time, functional aspects of cutaneous microcirculation of TT lesions. We have shown that TT skin lesions have a significant decrease of VD, PVD, PPV % and MFI in comparison to control skin, which reflects a significant impairment of capillary perfusion and deficient oxygenation in this region. An ultrastructural study demonstrated that cutaneous small vessels (capillaries) often possess an occluded lumen [6], due to endothelial cells hypertrophy, which can explain the poor cutaneous blood perfusion seen in TT lesion area. We believe that this microangiopathy may aggravate peripheral nerve degeneration caused by *M*. *leprae* infection. Decreased blood perfusion was also reported by our group in the other polar form of leprosy. In this recent study Treu and coworkers [22], using orthogonal polarization spectral imaging (OPS), showed that LL patients have a significant lower functional capillary density (number of perfused capillaries/mm^2^) in their skin compared to healthy controls.

In addition to functional capillary density, estimated by PVD, SDF allows the evaluation of FHI, which is the difference between the highest MFI and the lowest MFI divided by the mean MFI. The FHI reflects the perfusion heterogeneity and is a key determinant of oxygen extraction by tissues [16,25–27]. As we know, under normal circumstances, the microcirculation is able to regulate blood supply to tissues in order to meet their metabolic demand (i.e. autoregulation) and presents minimal flow heterogeneity. Normally, tissues better adapt to homogeneous (even slow) than heterogeneous blood flow. Decreasing functional capillary density and creating heterogeneous flow, oxygen diffusion distance increases, resulting in reduction of tissue oxygen extraction. Cells near perfused capillaries consume normal levels of oxygen. In contrast, cells distant from perfused capillaries do not receive enough oxygen to meet their needs and become hypoxic. As consequence, we can find hypoxic areas even in the presence of an elevated venous oxygen saturation [16,28,29] This phenomenon is characteristic of certain pathological states, such as sepsis. In sepsis occurs an impairment of tissue oxygen extraction despite normal or elevated oxygen delivery. Two hypotheses have been proposed to explain this phenomenon: (1) mitochondrial dysfunction, in which oxygen extraction is reduced as consequence of a decrease of oxygen utilization by mitochondria and (2) heterogeneity of capillary flow, which results in maldistribution of oxygen. Certain capillaries are undersupplied while others are oversupplied of oxygen. In other words, it means that the microvasculature loss the autoregulation capacity and fails to correct the maldistribution of blood flow and oxygen supply [27]. Evidences in the literature suggest that this incapacity is resultant of a signalization failure between arterioles and capillaries [30]. According to Ellis and coworkers [27], the decrease of oxygen extraction is rather a consequence of oxygen exchange surface loss and microvasculature inability to redistribute oxygen to tissues than mitochondrial dysfunction. In our study, despite the significant decrease in perfused vessel density in tuberculoid cutaneous lesions in comparison to control skin, FHI was not significantly different in the cutaneous lesion when compared to control skin, indicating that flow was not heterogeneous and probably oxygen extraction from tissues was not compromised.

In cutaneous lesions of TT there is a granulomatous infiltrate [31] without tissue edema formation. In general, edema formation occurs acutely in the curse of reactional states of leprosy (Type 1 reaction or reversal reaction and Type 2 reaction or erythema nodosum leprosum). These reactions are resultant of changes in the host immune response against antigenic determinants of *M*. *leprae* [32]. According to Wheate [33], the acute edema formation in tuberculoid leprosy patients occurs in and around reactional lesions, in eyelid and face. In our study, none of our patients were affected by any reactional states of leprosy, and therefore did not present edema that might have increased oxygen diffusion distance.

Concerning microvascular reactivity, we did not observe significant differences in endothelium -dependent and -independent vasodilatations between cutaneous lesion and healthy skin in TT patients, using LDF associated to ACh and SNP iontophoresis. In other words, microvascular function in TT skin lesions was preserved. Using the same technique, our group demonstrated that LL (the other polar form of Leprosy) caused a significant impairment of endothelial dependent and independent vasodilatations, which evidence an expressive microvascular dysfunction in these patients [22]. Ultrastructural studies of skin biopsies of LL patients revealed endothelial infection by *M*. *leprae* [6,34]. Moreover, Scollard and coworkers [35,36] demonstrated through electron microscopy that endothelial cells from epineural and perineural blood vessels and from human umbilical vein (HUVEC) were colonized by *M*. *leprae*. Since *M*. *leprae* has the capacity to infect endothelial cells and vasculature colonization by these *bacilli* may increase the risk of ischemia [36] and therefore endothelial damage. We believe that the significant microvascular dysfunction observed in LL patients could be consequence of endothelial cells infection and exposure of microvascular beds to chronic inflammation raised against *M*. *leprae*.

In order to evaluate the ultrastructure of microcirculation in different forms of leprosy Yajima and coworkers [6] reported that endothelial cells were hypertrophied and blood cells stagnated. The basement membrane of endothelial cells was associated with an increased number of inflammatory cells around blood vessels. The endothelial cells presented an extensive rough endoplasmic reticulum due to an increased protein synthesis. Moreover, another ultrastructural study involving blood vessels of nervous fibers demonstrated that endothelial cells extended to the lumen and, probably, for this reason, the vessel lumen was often closed. In addition, basement membrane of endothelial cells presented many folds and fingers, like protrusions [7].

The absence of intraendothelial *M*. *leprae*, shown in ultrastructural studies of TT skin and peripheral nerves biopsies [6,7], may explain the preserved endothelial function in cutaneous lesions of TT patients and accounts for discrepant results between the TT and LL patients, concerning endothelial function.

It was previously reported in the literature that *M*. *leprae* infection not only significantly affects somatic motor nervous fibers, but also autonomic ones [37]. Using laser Doppler velocimetry of fingertip microvascular blood flow, a reliable test to evaluate the vasomotor reflex (VMR), i.e. the vasoconstrictor response to sympathetic stimulus, such as inspiratory gasp, and sympathetic skin response (SSR), a method extensively used in clinical and experimental settings to assess sympathetic vasomotor function, this study [37] have demonstrated that the prevalence of VMR abnormalities and absent SSR were significantly higher in a heterogeneous cohort of Nepali leprosy patients than in controls. Years later, other study [38] have shown that abnormal VMR is also more prevalent in early diagnosed Brazilian Leprosy patients than in controls. Corroborating this observation, regarding VMR in leprosy patients, another work [39] demonstrated, by immunocytochemistry, that autonomic fibers that innervate blood vessels were compromised in TT patients. Based on these previous observations, we supposed that TT skin lesion could have a decreased contribution of the sympathetic component of flowmotion, but in our study, spectral analysis of flowmotion did not reveal any significant difference between TT cutaneous lesion and control skin concerning flowmotion sympathetic component. We presume that did not observe any significant difference between cutaneous lesion and control skin of these patients, concerning the sympathetic component of spectral analysis of flowmotion, due to low reproducibility of the method [40], which probably requires greater number of patients to reach statistical significance.

In order to exclude any confounding factors from our analysis we have considered ineligible for the study hypertensive, diabetic, obese, old and smoking subjects, since hypertension [41,42], diabetes mellitus [41,43], obesity [44], age [45,46] and tobacco [47–50] are factors that affect microvascular reactivity and vasomotion assessed by LDF. Moreover, we have decided not to include women in the study due to differences in gender hormones that may constitute an additional bias and lead to data misinterpretation. The rigid exclusion criteria allied to social stigma of TT patients contributed for the small number of included patients, which we considered the major study limitation.

## Conclusions

Our results suggest that tuberculoid leprosy cutaneous lesions have significant impairment of tissue perfusion and deficient oxygenation, which may aggravate peripheral nerve degeneration caused by *Mycobacterium leprae* infection.

## Supporting information

S1 Table Spectral analysis of flowmotion. (XLSX)

S2 Table Acetylcholine and sodium nitroprusside iontophoresis. (XLSX)

S3 Table Sidestream dark field imaging. (XLSX)

## References

[1] Walker SL, Lockwood DNJ. Leprosy. Clin Dermatol. 2007; 25:165–172. 10.1016/j.clindermatol.2006.05.012 17350495

[2] Britton WJ, Lockwood DN. Leprosy. Lancet. 2004; 363:1209–1219. 10.1016/S0140-6736(04)15952-7 15081655

[3] Agrawal A, Pandit L, Dalal M, Shetty JP. Neurological manifestations of Hansen’s disease and their management. Clin Neurol Neurosurg. 2005; 107:445–454. 10.1016/j.clineuro.2005.03.007 16202816

[4] Nath I, Saini C, Valluri VL. Immunology of leprosy and diagnostic challenges. Clin Dermatol. 2015; 33:90–98. 10.1016/j.clindermatol.2014.07.005 25432814

[5] Ridley DS, Jopling WH. Classification of leprosy according to immunity. A five-group system. Int J Lepr Other Mycobact Dis. 1966; 34:255–273. 5950347

[6] Yajima M, Murata J, Yamada N, Asano G. Ultrastructural observations of small blood vessels in leprosy patients. Nihon Rai Gakkai Zasshi. 1991; 60:121–127. 10.5025/hansen1977.60.121 1843224

[7] Kumar V, Narayanan RB, Malaviya GN. An ultrastructural study of blood vessels in peripheral nerves of leprosy patients: blood vessels in peripheral nerves. Nihon Rai Gakkai Zasshi. 1989; 58:179–184. 10.5025/hansen1977.58.179 2642166

[8] Cavender DE, Edelbaum D, Ziff M. Endothelial Cell Activation Induced by Tumor Necrosis Factor and Lymphotoxin. Am J Pathol 1989; 134:551–556. 2466402 PMC1879518

[9] Kraemer-Aguiar LG, Laflor CM, Bouskela E. Skin microcirculatory dysfunction is already present in normoglycemic subjects with metabolic syndrome. Metabolism. 2008; 57:1740–1746. 10.1016/j.metabol.2008.07.034 19013299

[10] Jonk AM, Houben AJ, Schaper NC, de Leeuw PW, Serné EH, Smulders YM, et al Meal-related increases in microvascular vasomotion are impaired in obese individuals: a potential mechanism in the pathogenesis of obesity-related insulin resistance. Diabetes Care. 2011;34: S342–S348. 10.2337/dc11-s240 21525480 PMC3632204

[11] Bouskela E, Grampp W. Spontaneous vasomotion in hamster cheek pouch arterioles in varying experimental conditions. Am J Physiol. 1992; 262:H478–H485. 10.1152/ajpheart.1992.262.2.H478 1539706

[12] Rossi M, Carpi A, Galetta F, Franzoni F, Santoro G. The investigation of skin blood flowmotion: a new approach to study the microcirculatory impairment in vascular diseases? Biomed Pharmacother. 2006; 60:437–442. 10.1016/j.biopha.2006.07.012 16935461

[13] Bertuglia S, Coluantoni A, Coppini G, Intaglietta M. Hypoxia or hyperoxia–induced changes in arteriolar vasomotion in skeletal muscle microcirculation. Am J Physiol. 1991; 260: H362–H372. 10.1152/ajpheart.1991.260.2.H362 1996682

[14] Turner J, Belch JJ, Khan F. Current concepts in assessment of microvascular endothelial function using laser Doppler imaging and iontophoresis. Trends Cardiovasc Med. 2008; 18:109–116. 10.1016/j.tcm.2008.02.001 18555183

[15] Tesselaar E, Sjöberg F. Transdermal iontophoresis as an *in-vivo* technique for studying microvascular physiology. Microvasc Res. 2011; 81:88–96. 10.1016/j.mvr.2010.11.002 21070791

[16] De Backer D, Hollenberg S, Boerma C, Goedhart P, Büchele G, Ospina-Tascon G, et al How to evaluate the microcirculation: report of a round table conference. Crit Care. 2007; 11: R101 10.1186/cc6118 17845716 PMC2556744

[17] Fitzpatrick TB. The validity and practicality of sun-reactive skin types I through VI. Arch Dermatol. 1988;124: 869–871. 10.1001/archderm.124.6.869 3377516

[18] Buss C, Kraemer-Aguiar LG, Maranhão PA, Marinho C, de Souza MD, Wiernsperger N, et al Novel findings in the cephalic phase of digestion: a role for microcirculation? Physiol Behav. 2012; 105:1082–1087. 10.1016/j.physbeh.2011.12.004 22197630

[19] Stefanovska A, Bracic M, Kvernmo HD. Wavelet analysis of oscillations in the peripheral blood circulation measured by laser Doppler technique. IEEE Trans Biomed Eng. 1999; 46:1230–1239. 10.1109/10.790500 10513128

[20] Serné EH, IJzerman RG, Gans RO, Nijveldt R, De Vries G, Evertz R, et al Direct evidence for insulin-induced capillary recruitment in skin of healthy subjects during physiological hyperinsulinemia. Diabetes. 2002; 51:1515–1522. 10.2337/diabetes.51.5.1515 11978650

[21] Kvernmo HD, Stefanovska A, Bracic M, Kirkebøen KA, Kvernebo K. Spectral analysis of the laser Doppler perfusion signal in human skin before and after exercise. Microvasc Res. 1998; 56:173–182. 10.1006/mvre.1998.2108 9828155

[22] Treu C, de Souza MDGC, Lupi O, Sicuro FL, Maranhão PA, Kraemer-Aguiar LG, et al Structural and functional changes in the microcirculation of lepromatous leprosy patients—Observation using orthogonal polarization spectral imaging and laser Doppler flowmetry iontophoresis. Plos One. 2017;12:e0175743 10.1371/journal.pone.0175743 28419120 PMC5395185

[23] Massey MJ, Shapiro NI. A guide to human in vivo microcirculatory flow image analysis. Crit Care. 2016; 20:35 10.1186/s13054-016-1213-9 26861691 PMC4748457

[24] Trzeciak S, Dellinger RP, Parrillo JE, Guglielmi M, Bajaj J, Abate NL, et al. Microcirculatory Alterations in Resuscitation and Shock Investigators. Early microcirculatory perfusion derangements in patients with severe sepsis and septic shock: relationship to hemodynamics, oxygen transport, and survival. Ann Emerg Med. 2007; 49:88–98. 17095120 10.1016/j.annemergmed.2006.08.021

[25] Humer MF, Phang PT, Friesen BP, Allard MF, Goddard CM, Walley KR, et al Heterogeneity of gut capillary transit times and impaired gut oxygen extraction in endotoxemic pigs. J Appl Physiol. 1996; 81:895–904. 10.1152/jappl.1996.81.2.895 8872661

[26] Farquhar I, Martin CM, Lam C, Potter R, Ellis CG, Sibbald WJ. Decreased capillary density *in vivo* in bowel mucosa of rats with normotensive sepsis. J Surg Res. 1996; 61:190–196. 10.1006/jsre.1996.0103 8769965

[27] Ellis CG, Bateman RM, Sharpe MD Sibbald WJ, Gill R. Effect of a maldistribution of microvascular blood flow on capillary O_2_ extraction in sepsis. Am J Physiol. 2002; 282:H156–H164. 10.1152/ajpheart.2002.282.1.H156 11748059

[28] De Backer D, Ospina-Tascon G, Salgado D, Favory R, Creteur J, Vincent JL. Monitoring the microcirculation in the critically ill patient: current methods and future approaches. Intensive Care Med. 2010;36:1813–1825. 10.1007/s00134-010-2005-3 20689916

[29] Tafner PFDA, Chen FK, Rabello R Filho, Corrêa TD, Chaves RCF, Serpa A Neto. Recent advances in bedside microcirculation assessment in critically ill patients. Rev Bras Ter Intensiva. 2017; 29:238–247. 10.5935/0103-507X.20170033 28977264 PMC5496759

[30] Tyml K, Yu J, McCormack DG. Capillary and arteriolar responses to local vasodilators are impaired in a rat model of sepsis. J Appl Physiol (1985). 1998; 84:837–844. 9480941 10.1152/jappl.1998.84.3.837

[31] Massone C, Belachew WA, Schettini A. Histopathology of the lepromatous skin biopsy. Clin Dermatol. 2015; 33:38–45. 10.1016/j.clindermatol.2014.10.003 25432809

[32] Naafs B, van Hees CL. Leprosy type 1 reaction (formerly reversal reaction). Clin Dermatol. 2016; 34:37–50. 10.1016/j.clindermatol.2015.10.006 26773622

[33] Wheate HW. Acute edema in leprosy. Int J Lepr. 1962; 30:388–394. 14000220

[34] Turkel SB, Van Hale HM, Rea TH. Ultrastructure of the dermal microvasculature in leprosy. Int J Lepr Other Mycobact Dis. 1982; 50:164–171. 6889577

[35] Scollard DM, McCormick G, Allen JL. Localization of *M*. *leprae* to endothelial cells of epineural and perineurial blood vessels and lymphatics. Am J Pathol. 1999; 154:1611–1620. 10329613 10.1016/S0002-9440(10)65414-4 PMC1866584

[36] Scollard DM. Association of *Mycobacterium leprae* with human endothelial cells *in vitro*. Lab Invest. 2000; 80:663–669. 10.1038/labinvest.3780069 10830776

[37] Wilder-Smith A, Wilder-Smith E. Electrophysiological evaluation of peripheral autonomic function in leprosy patients, leprosy contacts and controls. Int J Lepr Other Mycobact Dis. 1996; 64:433–440. 9030110

[38] Illarramendi X, Bührer-Sékula S, Sales AM, Bakker M I, Oliveira A, Nery JAC et al High prevalence of vasomotor reflex impairment in newly diagnosed leprosy patients. Eur J Clin Invest. 2005; 35: 658–665. 10.1111/j.1365-2362.2005.01554.x 16178886

[39] Karanth SS, Springall DR, Lucas S, Levy D, Ashby P, Levene MM, et al. Changes in nerves and neuropeptides in skin from 100 leprosy patients investigated by immunocytochemistry. J Pathol. 1989; 157:15–26. 10.1002/path.1711570104 2466111

[40] Cracowski JL, Minson CT, Salvat-Melis M, Halliwill JR. Methodological issues in the assessment of skin microvascular endothelial function in humans. Trends Pharmacol Sci. 2006; 27:503–508. 10.1016/j.tips.2006.07.008 16876881

[41] De Jongh RT, Serne EH, IJzerman RG, Stehouwer CD. Microvascular function: a potential link between salt sensitivity, insulin resistance and hypertension. J Hypertens. 2007; 25:1887–1893. 10.1097/HJH.0b013e32825e1db7 17762653

[42] Struijker-Boudier HA, Rosei AE, Bruneval P, Camici PG, Christ F, Henrion D, et al Evaluation of the microcirculation in hypertension and cardiovascular disease. Eur Heart J. 2007; 28:2834–2840. 10.1093/eurheartj/ehm448 17942581

[43] Quattrini C, Harris ND, Malik RA, Tesfaye S. Impaired skin microvascular reactivity in painful diabetic neuropathy. Diabetes Care. 2007; 30:655–659. 10.2337/dc06-2154 17327336

[44] De Jongh RT, Serne EH, IJzerman RG, Jorstad HT, Stehouwer CD. Impaired local microvascular vasodilatory effects of insulin and reduced skin microvascular vasomotion in obese women. Microvasc Res. 2008; 75:256–262. 10.1016/j.mvr.2007.08.001 17920639

[45] Li L, Mac-Mary S, Marsaut D Sainthillier JM, Nouveau S, Gharbi T, et al. Age-related changes in skin topography and microcirculation. Arch Dermatol Res. 2006; 297:412–416. 10.1007/s00403-005-0628-y 16328340

[46] Li L, Mac-Mary S, Sainthillier JM, Nouveau S, de Lacharriere O, Humbert P. Age-related changes of the cutaneous microcirculation *in vivo*. Gerontology. 2006; 52:142–153. 10.1159/000091823 16645294

[47] Monfrecola G, Riccio G, Savarese C, Posteraro G, Procaccini EM. The acute effect of smoking on cutaneous microcirculation blood flow in habitual smokers and nonsmokers. Dermatology. 1998; 197:115–118. 10.1159/000017980 9732157

[48] Pellaton C, Kubli S, Feihl F, Waeber B. Blunted vasodilatory responses in the cutaneous microcirculation of cigarette smokers. Am Heart J. 2002; 144:269–274. 10.1067/mhj.2002.123842 12177644

[49] IJzerman RG, Serne EH, van Weissenbruch MM, De Jongh RT, Stehouwer CD. Cigarette smoking is associated with an acute impairment of microvascular function in humans. Clin Sci (Lond). 2003; 104:247–252. 12605581 10.1042/CS20020318

[50] Edvinsson ML, Andersson SE, Xu CB, Edvinsson L. Cigarette smoking leads to reduced relaxant responses of the cutaneous microcirculation. Vasc Health Risk Manag. 2008; 4:699–704. 10.2147/vhrm.s2285 18827920 PMC2515430

